# Women's growing desire to limit births in sub-Saharan Africa: meeting the challenge

**DOI:** 10.9745/GHSP-D-12-00036

**Published:** 2013-03-21

**Authors:** Lynn M Van Lith, Melanie Yahner, Lynn Bakamjian

**Affiliations:** a Johns Hopkins Bloomberg School of Public Health Center for Communication Programs, Baltimore, MD, USA; b EngenderHealth, New York, NY, USA; c International Health and Development Consultant

## Abstract

Demographic and Health Survey data from 18 countries were analyzed to better understand the characteristics of women wishing to limit childbearing. Demand for limiting (14% of all women) is less than that that for spacing (25%) but is still substantial. The mean “demand crossover age” (the average age at which demand to limit births begins to exceed demand to space) is generally around age 33, but in some countries it is as low as 23 or 24. Young women often intend to limit their births, contrary to the assumption that only older women do. Large numbers of women have exceeded their desired fertility but do not use family planning, citing fear of side effects and health concerns as barriers. When analysis is restricted to married women, demand for limiting nearly equals that for spacing. Many women who want no more children and who use contraception, especially poor women and those with less education, use less effective temporary contraceptive methods. A sizable number of women in sub-Saharan Africa—nearly 8 million—have demand for limiting future births. Limiting births has a greater impact on fertility rates than spacing births and is a major factor driving the fertility transition. Family planning programs must prepare to meet this demand by addressing supply- and demand-side barriers to use. Meeting the growing needs of sub-Saharan African women who want to limit births is essential, as they are a unique audience that has long been overlooked and underserved.

## Abstract (precis)

Contrary to conventional wisdom, many sub-Saharan African women—often at young ages—have an unmet need for family planning to limit future births, and many current limiters do not use the most effective contraceptive methods. Family planning programs must improve access to a wide range of modern contraceptive methods and address attitudinal and knowledge barriers if they are to meet women's needs.

## INTRODUCTION

While contraceptive use has risen to relatively high levels in many areas of Asia and Latin America and the Caribbean, it remains low in much of sub-Saharan Africa. Only about 1 in 4 women of reproductive age in Africa use a modern method of family planning,^1^ and this proportion is substantially lower in many countries of the region. These numbers, however, do not indicate a lack of interest in family planning among women in the region.

Birth spacing is a commonly used concept in family planning programs in Africa—a concept that is often tied to a health rationale for contraception.^2^ However, less of the literature focuses on the group of women in sub-Saharan Africa with the desire to limit (or end) childbearing, even though the proportion of limiters exceeds spacers in several countries in Africa.^2^

Trend data suggest that the proportion of women in sub-Saharan Africa who want to limit rather than postpone childbearing is rising steadily.^3^ Increases in the demand for contraception, particularly in eastern and southern Africa, stem primarily from the rising proportion of women who wish to cease rather than postpone childbearing.^3^^,^^4^ (See box for definitions of demand and unmet need.) Increasing use of contraception among these women will reduce high-risk, high-parity births, thereby contributing to the reduction of maternal mortality.^5^ Further, meeting the needs of this group is important for 2 reasons:

1. Birth-limiting behavior has a greater impact on fertility rates than does birth spacing.^6^^,^^7^
2. Such behavior has been a major factor in driving the fertility transition in Africa.^8^

Contraceptive use to limit births has a greater impact on fertility rates than using contraception to space births.

**Box. Key Definitions**

**Demand for family planning** is the desire or motivation of women (or couples) to control their future fertility. Demand for spacing births exists when women would like to wait 2 or more years before their next birth, while demand for limiting exists when women say that they do not want any more children. Demand for family planning consists of both met need (current use of family planning) and unmet need.

**Unmet Need**

Unmet need for family planning*—the percentage of women who do not want to become pregnant but are not using contraception—is the measure most commonly used to indicate potential demand. Couples with an unmet need for family planning are subdivided into 2 groups:

Women with an **unmet need for spacing** are those who are fecund (able to become pregnant) but are not using family planning and who:

Want to postpone their next birth for 2 or more years;
Are pregnant/postpartum amenorrheic and say that their current pregnancy/last birth was mistimed;
Are unsure whether they want another child; or
Want another child but are unsure when.

Women with an **unmet need for limiting** are those who are fecund (able to become pregnant) but are not using family planning and who:

- Say they do not want another child;
- Are pregnant/postpartum amenorrheic and say that their current pregnancy/last birth was unwanted, irrespective of whether they say they want another child in the future; or
- Are undecided about whether they want another child in the future.

*MEASURE DHS recently standardized its definition of unmet need for family planning across all country surveys, including the decision to no longer include contraceptive calendar data. For countries that had previously collected calendar data consistently, revised unmet need figures are higher than under the previous definition. For countries that collected calendar data inconsistently, unmet need trends changed after revision but are now more accurately represented. Among the 18 countries with DHS data analyzed in this study, 11 did not collect calendar data (Benin, Cameroon, Ghana, Lesotho, Madagascar, Namibia, Rwanda, Senegal, Swaziland, Uganda, Zambia); 1 used calendar data consistently (Zimbabwe); and 3 (Kenya, Malawi, and Tanzania) used calendar data in some surveys but not in the most recent ones, and no changes in unmet need were made for the most recent survey. The majority of the countries analyzed were therefore not affected by the definition change. For more information, see reference 12.

Fertility intention is an important predictor of subsequent reproductive behavior, and contraceptive use intentions are an even better predictor, particularly among women who want to limit future births.^9-11^ Limiters may have a stronger desire to avoid pregnancy than do spacers. If a spacer has a birth earlier than planned, that birth presumably was still desired, although perhaps mistimed, and would have occurred regardless, whereas an unintended pregnancy for a limiter directly adds to the fertility rate overall.

Meeting women's reproductive intentions in the context of informed choice enables them to have the number of children they desire, improves the health and well-being of both women and their families, and ultimately affects macro-level health and development indicators. In this article, we examine Demographic and Health Surveys (DHS) data in a sample of sub-Saharan African countries to better understand the characteristics of women who intend to limit future births and discuss how programs may better serve them to reduce unmet need in sub-Saharan Africa.

## DATA AND METHODS

This analysis focuses on DHS datasets from 18 countries in sub-Saharan Africa that were surveyed between 2004 and 2010 (Table 1). The DHS is a nationally representative household survey that explores, among other indicators, women's demand for and use of contraception; the surveys are led by ICF Macro/MEASURE DHS, in collaboration with local institutions.^13^

All sub-Saharan African countries with a DHS after the year 2000 were eligible for inclusion in the analysis. We selected countries based on the presence of a sufficient number of users (25 or more) of each of the 4 contraceptive method categories included in the analysis, to allow for sufficient sample sizes. We also included high-population countries (for example, Ethiopia, the Democratic Republic of Congo [DRC]), to ensure that the analyses are representative of much of the region's population. Fourteen countries in sub-Saharan Africa were excluded due to small sample size.

**Table 1.** Countries and Survey Years Included in the Analysis

Country	Survey Year
Benin	2006
Cameroon	2004
Democratic Republic of Congo	2007
Ethiopia	2011
Ghana	2009
Kenya	2008/9
Lesotho	2004 and 2009*
Madagascar	2009
Malawi	2010
Namibia	2007
Nigeria	2008
Rwanda	2010
Senegal	2010–11
Swaziland	2007
Tanzania	2010
Uganda	2006
Zambia	2007
Zimbabwe	2010–11

* The 2004 Lesotho DHS was used for data that were not included in the 2009 DHS.

We used STATA Version 9 and SPSS Version 20 to analyze the individual datasets for each country. DHS data for the 18 analysis countries were also explored through StatCompiler, particularly for common indicators such as contraceptive prevalence. The research presented here is part of a larger global analysis of DHS data that explored characteristics of users of various types of family planning methods, as well as of nonusers.

Data were weighted, and women using contraception were categorized as users of short-acting methods, long-acting reversible contraceptives (LARCs), permanent methods, or traditional methods. The LARC and permanent method categories were consistent across countries; **LARCs** comprised intrauterine devices (IUDs) and hormonal implants while **permanent methods** consisted of female and male sterilization. Since use of male sterilization is very low or nonexistent in all of the analysis countries, nearly all permanent method use consists of female sterilization.

**Short-acting methods** consisted of the pill, male and female condoms, the Standard Days Method®, diaphragms, spermicides, and injectables. (Although injectables are effective for up to 3 months, we classified them as a short-acting method, as is the norm.) The mix of short-acting methods varied slightly by country, mostly based on the presence or absence of female condoms and spermicides.

The level of detail provided in the dataset for **traditional methods** also varied by country, but these methods consisted primarily of withdrawal, periodic abstinence, and folk methods. For each country, whichever short-acting methods and traditional methods were present in the dataset were included in the respective categories. The analysis included all women of reproductive age (ages 15 to 49). When averages across countries are presented, data are weighted by the number of women of reproductive age in the country.

## RESULTS

### Demand for Limiting Is Strong in sub-Saharan Africa, Even Among Younger Women

Although fertility desires are generally high in the region, demand for limiting (met and unmet need) is strong as well:

- Among all women of reproductive age in the analysis countries, more have a demand to space births (25%) than to limit (14%), using an average weighted by population size of women of reproductive age. However, among married women, demand for limiting nearly equals that for spacing in the analysis countries (26% versus 31%, respectively).
- 37% of all demand for family planning is for limiting.
- An average of 9% of women across the 18 countries reported that they had wanted no more children at the time of their last birth, ranging from 4% in Benin to 37% in Swaziland.

Typically, demand for birth spacing exceeds that for birth limiting among younger women, while older women—having achieved their desired family size—more often have a demand for limiting births. On average, limiters are a decade older than spacers (average age of 37 versus 27, respectively).

The **“demand crossover age”** is the average age at which demand to limit future births begins to exceed demand to space births. This occurs when women reach their desired family size and wish to cease childbearing.

**FIGURE 1:**
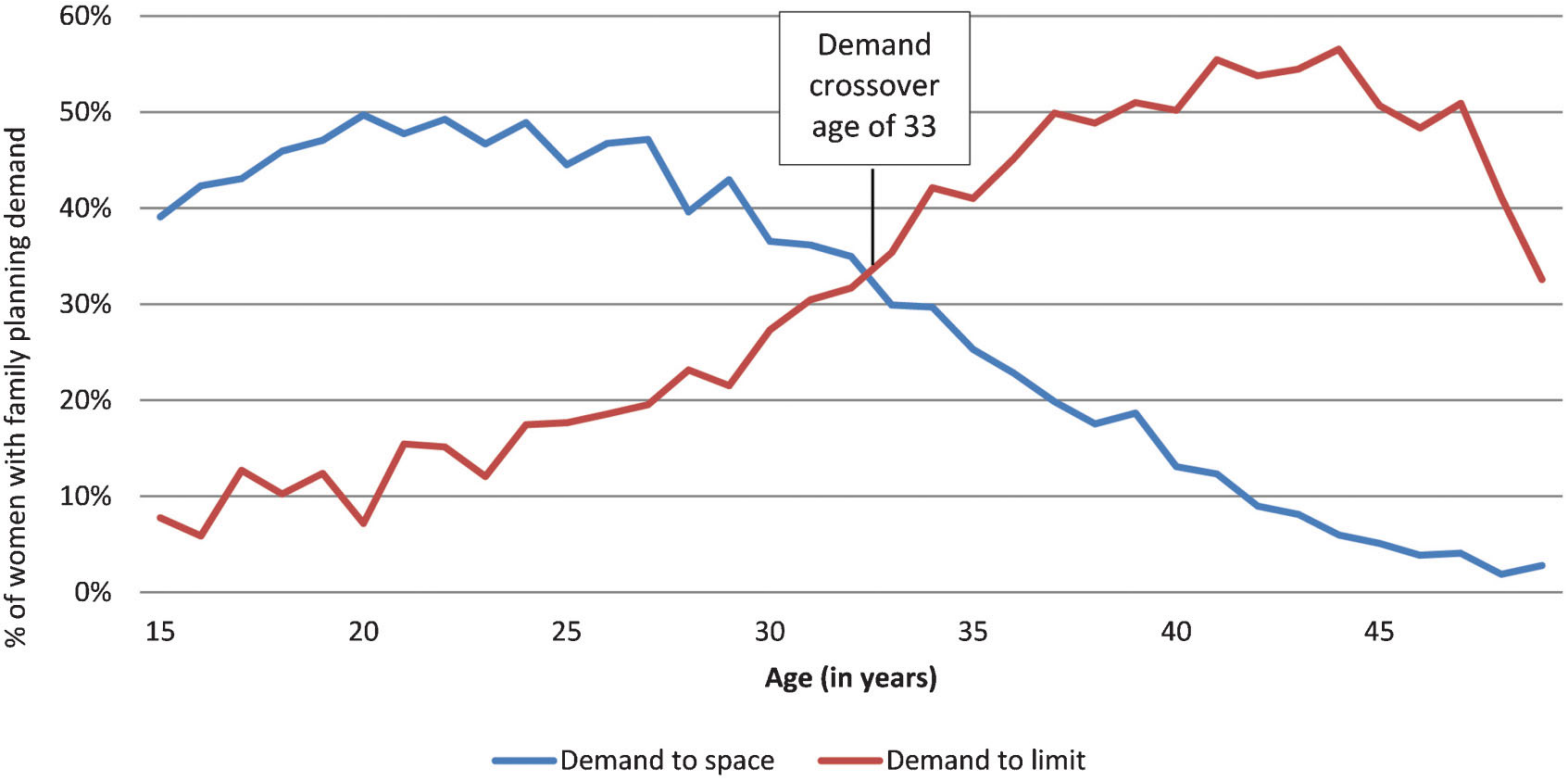
Demand for Spacing and Limiting Births,^a^ by Age ^a^ Averages weighted by population of women of reproductive age for all 18 analysis countries

On average, in the analysis countries, demand to limit begins to exceed demand to space at age 33 (Figure 1); however, in some countries, particularly in Southern Africa, the demand crossover age is considerably lower. For example, in Swaziland, the average age at which the demand to limit meets or exceeds that to space is 23; in Lesotho, it is 24 (Table 2).

**Table 2.** Demand Crossover Age: Mean Age at Which Demand for Limiting Future Births Meets or Exceeds Demand for Spacing Births

Country	Age
Swaziland	23
Lesotho	24
Namibia	28
Malawi	29
Kenya	31
Madagascar	31
Rwanda	31
Ethiopia	32
Zimbabwe	32
Uganda	33
Benin	34
Tanzania	34
Cameroon	35
Zambia	35
Ghana	36
Nigeria	36
Democratic Republic of Congo	38
Senegal	38

Demand to limit births begins to exceed demand to space births, on average, at age 33.

While demand for limiting is often greatest among older women (35 and older), our analysis shows that young women also have a demand to limit.

- In Swaziland, for example, 44% of women ages 25–29 have a demand for limiting, while 24% have a demand for spacing.
- Similarly, among women ages 25–29, 35% in Lesotho, 30% in Namibia, 26% in Kenya, and 13% in Ethiopia have a demand for limiting.

In some countries, substantial demand for limiting births exists even among the youngest women, with 22% of women between the ages of 20–24 in Namibia and 29% in Swaziland having a demand for limiting.

Substantial demand for limiting births exists even among the youngest women in some countries.

Based on current populations of women of reproductive age and unmet need data from the most recent DHS, we estimate that in 2012, more than 7.8 million women in the 18 countries included in this analysis have an unmet need for limiting future births.

An estimated 8 million women in 18 sub-Saharan African countries have an unmet need for limiting births.

### Use of Family Planning for Limiting Is Sizable

A sizable proportion of all women in every country reported a demand for limiting births, ranging from 8% in Senegal to 35% in Swaziland (Table 3). In one-third of the countries studied (6 of 18), demand to limit exceeds demand to space. In Swaziland, for example, 35% have a demand to limit, compared with 16% who have a demand to space births.

**Table 3.** Met and Unmet Need and Total Demand for Spacing and Limiting Births, by Country

Country	Demand to limit (%)	Using to limit (%)	Unmet need to limit (%)	Demand to space (%)	Using to space (%)	Unmet need to space (%)
Benin	14.8	5.4	9.4	26.0	11.8	14.2
Cameroon	10.6	6.4	4.2	29.9	19.6	10.3
Democratic Republic of Congo	9.3	5.8	3.5	27.7	14.3	13.4
Ethiopia	14.1	8.5	5.6	21.5	11.1	10.4
Ghana	14.9	7.2	7.7	27.4	12.2	15.2
Kenya	26.8	19.0	7.8	21.4	13.0	8.4
Lesotho	30.3	22.8	7.5	19.6	13.2	6.4
Madagascar	22.0	15.8	6.2	24.3	15.9	8.4
Malawi	28.0	19.8	8.2	25.8	15.6	10.2
Namibia	30.9	25.9	5.0	25.1	20.8	4.3
Nigeria	8.2	4.4	3.8	22.9	11.0	11.9
Rwanda	20.8	16.0	4.8	18.0	12.6	5.4
Senegal	8.0	3.0	5.0	21.4	6.6	14.8
Swaziland	35.0	26.4	8.6	16.0	11.5	4.5
Tanzania	17.5	10.8	6.7	29.6	18.0	11.6
Uganda	19.9	9.6	10.3	26.1	10.0	16.1
Zambia	17.6	11.4	6.2	30.3	18.5	11.8
Zimbabwe	22.3	18.6	3.7	27.6	22.7	4.9

Although use of family planning for spacing exceeds that for limiting in many analysis countries, limiters comprise a majority of family planning use in more than one-third of the countries analyzed. The percentage of women using contraception to limit births (calculated by dividing the percentage using a method to limit births by the overall percentage using either to space or to limit births in Table 3) is highest in Swaziland (70%), Lesotho (63%), Kenya (59%), Namibia (55%), Malawi and Rwanda (56%), and Madagascar (50%).

The overwhelming majority of family planning users in older age groups are limiters, ranging from 76% of family planning users ages 45–49 in the DRC to 99% in Zambia. However, many women in younger age groups use family planning for limiting as well: 45% of Malawian family planning users, 33% of Ethiopian family planning users, and 20% of Ghanaian users ages 25–29 are limiters.

### Limiters Use Short-Acting Methods More Than LARCs and Permanent Methods

**FIGURE 2:**
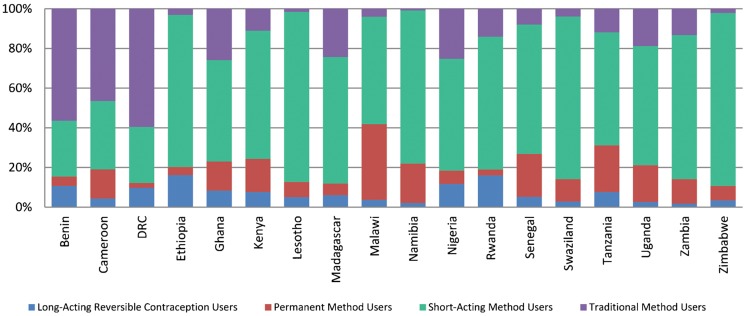
Method Mix Among Women Using Contraception to Limit Births

Contraceptives vary widely in their effectiveness, with traditional and short-acting methods having lower rates of effectiveness during typical use than long-acting or permanent methods. Typical-use failure rates (the percentage of women experiencing an unintended pregnancy during the first year of typical use) for short-acting methods range from 6% (Depo-Provera injectables) to 28% (spermicides), while failure rates for traditional methods can be as high as 22% (withdrawal). In contrast, all long-acting and permanent methods have failure rates of less than 1%.^14^ In all of the countries included in our analysis, family planning users who would prefer to stop childbearing were more likely to use short-acting or traditional methods than the more effective LARCs and permanent methods (Figure 2):

- On average, 80% of limiters in the analysis countries use a short-acting or traditional method; 95% of spacers use such a method.
- In 15 of the 18 analysis countries, more than half of women using family planning for limiting rely on short-acting methods.
- Variation between countries does exist. In Malawi, for example, 38% of limiters use permanent methods, as do 23% of limiters in Tanzania.

Contraceptive users who want to limit births are more likely to use short-acting or traditional methods than more effective long-acting or permanent methods.

In 8 of the countries, less than 10% of the method mix was attributable to LARCs and permanent methods. Among family planning users in the selected countries, short-acting methods, particularly injectables, are the most commonly used methods, and LARCs and permanent methods generally constitute a small fraction of the method mix. However, this low number of women that use LARCs and permanent methods may represent only a small proportion of the potential market for these methods. The data show that many more women hope to use a LARC or a permanent method in the future. Furthermore, in 7 of the countries in this review, more women reportedly intend to use a LARC or a permanent method than current users of these methods.

### Many Limiters Have Met or Exceeded Ideal Parity

**FIGURE 3:**
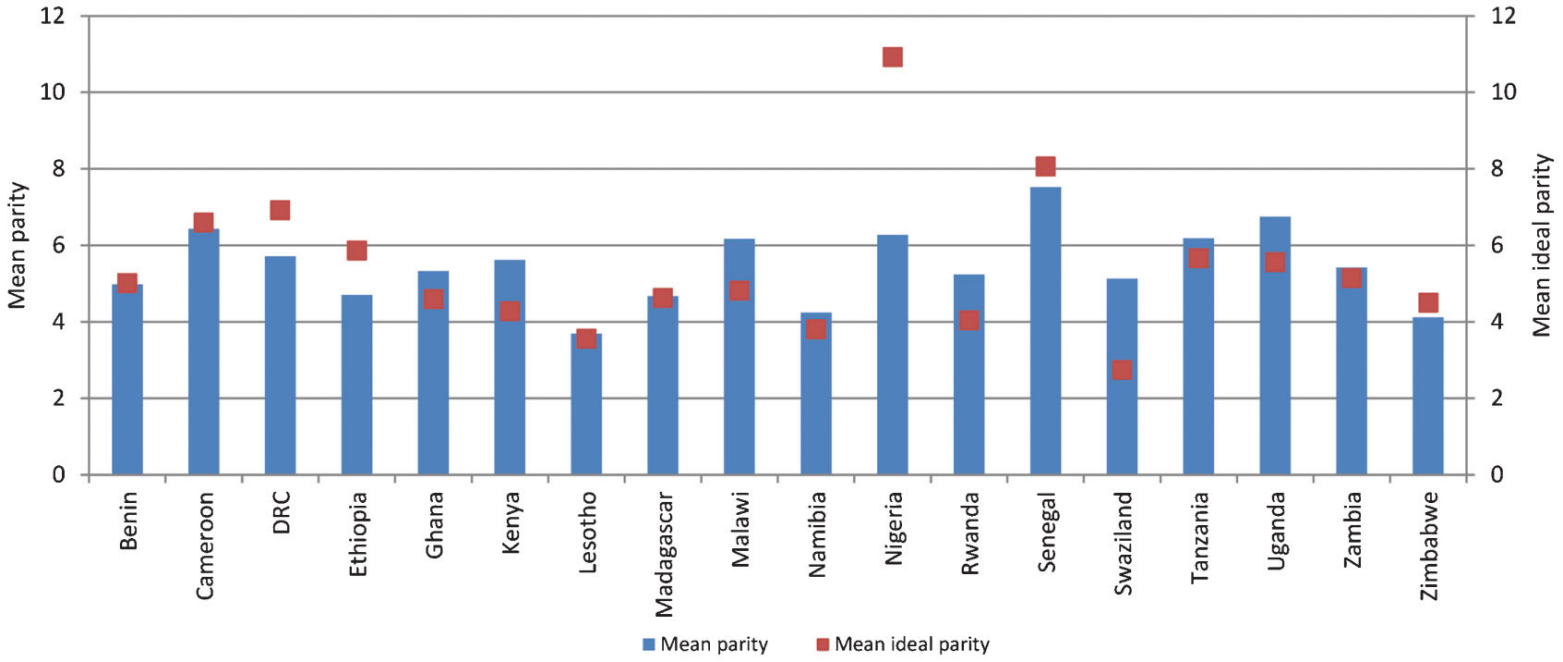
Mean Parity and Mean Ideal Parity Among Users of Permanent Contraceptive Methods

On average, 28% of women with a demand to limit have met their ideal parity and 30% have exceeded it. In Rwanda and Swaziland, more than half of limiters have exceeded their ideal parity (54% and 52%, respectively). This contrasts sharply with spacers, of whom 5% have met their ideal parity. In the 18 countries studied, a large proportion of women have reached or exceeded their ideal parity:

- In 15 countries, more than one-quarter of permanent method users have exceeded their ideal parity.
- In 5 of the 15 countries, more than half have exceeded their ideal parity. For example:
  - In Malawi—a country where a large proportion of the modern method mix is attributable to permanent methods (23%)—57% of women using sterilization have had more than their ideal number of children. In Swaziland, 69% of permanent method users have exceeded their ideal parity.
  - In Kenya, Malawi, Rwanda, Swaziland, and Uganda, permanent method users have exceeded their ideal parity by an average of more than one birth (Figure 3).

Many permanent method users have had more than their ideal number of children.

In contrast, in all but 3 countries (Kenya, Rwanda, and Swaziland), the majority of short-acting method users have not yet reached their ideal parity. However, many will have done so after their next birth.

### Fewer Poor Women Use Contraception Than Wealthy Women

**FIGURE 4:**
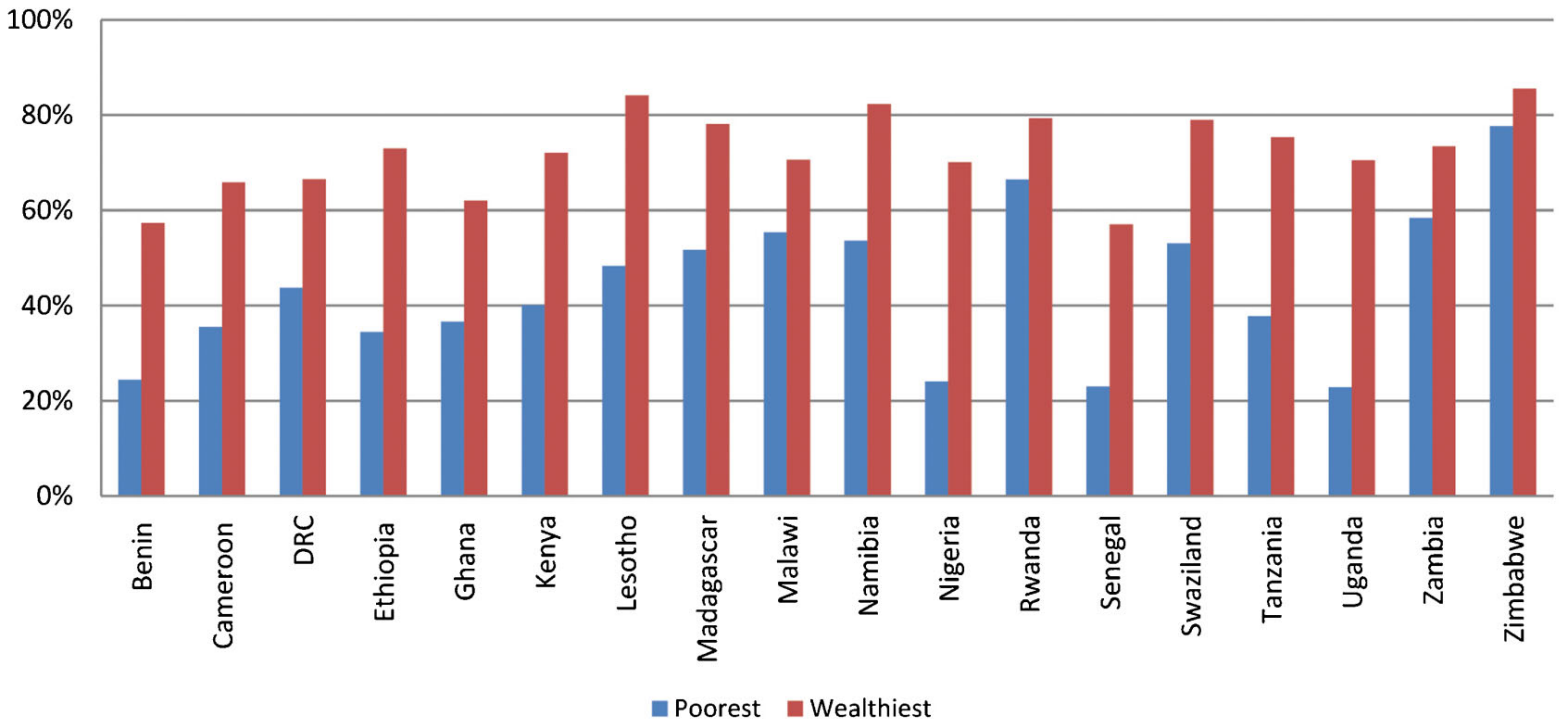
Percent of Demand for Limiting Satisfied Among the Poorest and Wealthiest Quintiles

About 12% of women in the wealthiest quintile used family planning for limiting, compared with only 5% among those in the poorest quintile. Indeed, in some countries, the differences between the wealthiest and poorest quintiles are striking. In Namibia, for example, 30% of the wealthiest women use family planning for limiting, while just 16% of the poorest do so, and 15% of the wealthiest Ugandan women use family planning to limit, compared with just 4% of the poorest. On average, in the 18 analysis countries, 74% of demand for limiting among the wealthiest women is *satisfied*, while only 40% of the poorest women's demand is satisfied. In some countries, these differences are even more considerable (Figure 4).

### Contraceptive Use Varies by Education Despite Nearly Equal Demand

Although women with the highest and lowest levels of education have nearly equal demand for limiting (14.4% versus 14.2%, respectively), disparities in education affect family planning use for limiting. Women who have completed a higher education are nearly twice as likely to use family planning for limiting (12%) than are women who have received no formal education (7%). Further, the most educated women have less than half the level of unmet need for limiting (6%) as their counterparts who have no formal education (14%). While 80% of demand for limiting is satisfied among women who have completed a higher education, only 40% is satisfied among women who have no education.

### Barriers to Use Include Fear of Side Effects and Health Concerns

In our analysis, women with an unmet need for limiting who did not intend to use family planning in the future most often cited fear of side effects (17%) or health concerns (12%) as their primary reason for not using contraception. (Analysis on this particular indicator excluded Ethiopia, Lesotho, Malawi, Rwanda, Senegal, Tanzania, and Zimbabwe, as this question was not asked in the most recent DHS.) In addition, despite an expressed desire to not become pregnant again, 13% reported that they themselves are opposed to family planning use. Infrequent sex was also cited by many limiters for their lack of intent to use family planning in the future (15%). The primary reasons for not using contraception do not differ vastly between limiters and spacers: fear of side effects (18%) is the reason most often cited for nonuse among spacers. Spacers, however, more often report that they (17%) or their husband (10%) are opposed to family planning use.

While lack of knowledge about or access to methods is a barrier to use, it was not articulated as a primary barrier among limiters who did not use contraception; an average of 5% said that they did not know of a method, 1% said they knew no source, and 2% cited lack of access or cost barriers.

Even current users can lack information about family planning: 54% of pill users, 47% of female sterilization users, 45% of injectable users, 29% of IUD users, and 25% of implant users reported that they were not informed about potential side effects or other problems associated with their method.

Many contraceptive users report that they were not informed about potential side effects with their method or about other methods that they could use.

Our results also show that an average of 43% of current pill users and 37% of injectable users in the analysis countries reported that they were not informed about other methods that they could use. This reported lack of information is not limited to users of short-acting methods; on average, 51% of female sterilization users, 34% of implant users, and 24% of IUD users reported that they were not informed, at the specific time the service was provided, about other methods that they could use.

## DISCUSSION

Whether women use a family planning method often depends on the fit between their fertility preferences and the choices available.^11^ Making more contraceptive options available in a program's method mix unmistakably raises contraceptive prevalence^15^ and helps to ensure informed choice. While women undoubtedly should be able to use their method of choice, it is well known that many women in the countries under review here have limited options, given pervasive knowledge-related, access-related, and societal barriers, as well as resource constraints.

Expanding the method mix improves contraceptive use.

While access was not mentioned as a primary barrier to use in the DHS data used in our analysis, it may still be a significant issue. Given that poorer women are less likely to use contraception than wealthier women, quality information and services may not be as available in poor or hard-to-reach areas. Further, since many women have exceeded their desired parity, we question whether family planning options are readily offered and available to postpartum women when they may need these methods the most. A lack of information about side effects and method options raises another concern about the quality of counseling and client-provider interaction.

Our analysis suggests that many sub-Saharan African women with an unmet need for limiting future births continue to fear side effects and cite health concerns as primary reasons for their lack of intention to use family planning in the future. These barriers, coupled with societal and familial opposition, are part of the complex nature of influences that drive contraceptive decision making. Family planning programs must address these multiple domains of influence. Evidence demonstrates that exposure to social and behavior change communication messages has a positive effect on family planning ideation (including knowledge of contraceptive methods, spousal communication, and favorable attitudes); on contraceptive use; and on the intention to use a method in the future.^16-19^ Exposure to such messages, coupled with proven supply-side approaches, is needed.

Fear of side effects and health concerns continue to be major barriers to contraceptive use in sub-Saharan Africa.

A limitation of this study is that the countries included in the analysis represented only a subset of sub-Saharan African countries that had recent DHS surveys, albeit the 18 countries included in the study represent the large majority of the population in the region. Additionally, not all possible questions are included in every DHS; some relevant questions were omitted from some of the surveys analyzed.

Further, some have questioned the ability of DHS survey questions to truly capture intention to limit, particularly given the ambiguity that many women may feel when asked such questions.^8^^,^^20^ Others argue that because African life is exceedingly uncertain, parents may neither deem the number of children born as important nor conceptualize an end to childbearing with any degree of meaning.^21^ Finally, the DHS, as it collects information via household surveys, is a rich source of information about demand for family planning, but it provides little information about access to and availability of methods or about quality of services. While the DHS has its limitations, it still provides the best measures available to compare fertility desires across multiple countries in a meaningful way. However, we recognize the usefulness of having qualitative data to elucidate women's fertility intentions in more depth.

Although differences between countries are large and require context-specific responses, what is clear is that fertility is likely to continue to decline in sub-Saharan Africa.^22^ If this trend holds, more and more sub-Saharan African women will want to limit childbearing, which will require advance preparation from family planning programs, with supply- and demand-side inputs, as well as policy and budgetary commitments.

We have tried to understand the profile and needs of women who want to limit future childbearing in several countries across sub-Saharan Africa; further research is needed to uncover and appreciate the many barriers that women face in meeting their reproductive intentions, so that program managers and policy makers may, in turn, develop more effective and culturally relevant strategies to support women in their contraceptive decision making.

Facilitating the ability of women and couples to make informed choices about the number, timing, and spacing of their births supports a fundamental human right that must always be at the core of family planning programs.^23^ While much remains to be learned about fertility desires across sub-Saharan Africa, let us not assume that because fertility decline on the continent has occurred slowly compared with other parts of the globe that desires to limit family size are antithetical in Africa.^24^ On the contrary, many sub-Saharan African women are interested not only in spacing births but also in limiting births, and many are already taking action to limit their fertility.

## CONCLUSION

Our findings confirm that women at younger ages have a significant unmet need for limiting, but for a multitude of reasons they are either unable to or choose not to use family planning to avoid pregnancy. Through efforts to expand knowledge of and access to highly effective modern methods, more women may use them to meet their reproductive health needs. We argue for placing as much attention on the growing number of women with the intention to end childbearing as on those who want to space births; the consequences of unintended pregnancies among those who wish to limit are as detrimental as those among women whose pregnancies are spaced too closely together. Women who want to limit future childbearing are a unique audience that has long been overlooked and underserved. Family planning programs must therefore address the needs of both spacers *and* limiters to meet the needs of women in sub-Saharan Africa.
